# Alterations in osteoclast function and phenotype induced by different inhibitors of bone resorption - implications for osteoclast quality

**DOI:** 10.1186/1471-2474-11-109

**Published:** 2010-06-01

**Authors:** Anita V Neutzsky-Wulff, Mette G Sørensen, Dino Kocijancic, Diana J Leeming, Morten H Dziegiel, Morten A Karsdal, Kim Henriksen

**Affiliations:** 1 Nordic Bioscience A/S, Herlev, DK-2730, Denmark; 2 HS Blodbank, Copenhagen University Hospital, Copenhagen, Denmark

## Abstract

**Background:**

Normal osteoclasts resorb bone by secretion of acid and proteases. Recent studies of patients with loss of function mutations affecting either of these processes have indicated a divergence in osteoclastic phenotypes. These difference in osteoclast phenotypes may directly or indirectly have secondary effects on bone remodeling, a process which is of importance for the pathogenesis of both osteoporosis and osteoarthritis. We treated human osteoclasts with different inhibitors and characterized their resulting function.

**Methods:**

Human CD14 + monocytes were differentiated into mature osteoclasts using RANKL and M-CSF. The osteoclasts were cultured on bone in the presence or absence of various inhibitors: Inhibitors of acidification (bafilomycin A1, diphyllin, ethoxyzolamide), inhibitors of proteolysis (E64, GM6001), or a bisphosphonate (ibandronate). Osteoclast numbers and bone resorption were monitored by measurements of TRACP activity, the release of calcium, CTX-I and ICTP, as well as by counting resorption pits.

**Results:**

All inhibitors of acidification were equally potent with respect to inhibition of both organic and inorganic resorption. In contrast, inhibition of proteolysis by E64 potently reduced organic resorption, but only modestly suppressed inorganic resorption. GM6001 alone did not greatly affect bone resorption. However, when GM6001 and E64 were combined, a complete abrogation of organic bone resorption was observed, without a great effect on inorganic resorption. Ibandronate abrogated both organic and inorganic resorption at all concentrations tested [0.3-100 μM], however, this treatment dramatically reduced TRACP activity.

**Conclusions:**

We present evidence highlighting important differences with respect to osteoclast function, when comparing the different types of osteoclast inhibitors. Each class of osteoclast inhibitors will lead to different alterations in osteoclast quality, which secondarily may lead to different bone qualities.

## Background

Bone remodeling is an important and integrated part of the processes controlling the age and quality of the bone matrix [1]. Bone remodeling is mediated by two cell types: Osteoclasts, which are the only cells with the ability to degrade the calcified bone matrix, and osteoblasts, which are bone-forming cells [1]. Bone resorption is always followed by bone formation in a tightly balanced manner referred to as coupling [2,3]. Recent studies in humans with mutations altering the ability of osteoclasts to perform bone resorption have indicated that osteoclasts, in addition to their role in bone resorption, play important roles in orchestrating bone formation [3-6]. Recent studies have furthermore demonstrated that osteoclasts on plastic (non-resorbing osteoclasts) secrete signals which induce bone formation [7,8]. It may therefore be speculated that the phenotype of osteoclasts is highly important for the cell-to-cell signaling between osteoclasts and osteoblasts, and may have secondary effects on bone formation during bone remodeling [4,5].

Bone resorption is initiated by osteoclasts forming a sealing zone and ruffled border at their interface with bone. Through the ruffled border the osteoclasts secrete hydrochloric acid [9] to dissolve the inorganic matrix, for which low pH is required. The secretion of protons from osteoclasts is an active process, which requires activity of the osteoclast-specific vacuolar ATPase (V-ATPase) containing the a3 subunit [10,11]. Chloride transport through the ruffled border occurs via the chloride-proton antiporter ClC-7 [12-15], and thereby electroneutrality across the membrane is maintained. Elimination of either of the two process leads to complete loss of acid secretion and hence neither the inorganic matrix nor the organic matrix are dissolved [15]. Loss of function of either the osteoclast-specific V-ATPase a3 subunit or ClC-7 leads to the osteopetrotic human disorders: autosomal recessive osteopetrosis (ARO) or autosomal dominant osteopetrosis type II (ADOII) [12,16-19]. Osteopetrosis in both man and mice is characterized by a high bone mass phenotype, due to lack of osteoclast function as described above [10-15,20].

In order to generate protons, the presence of carbonic anhydrase II (CAII) is essential. It catalyzes the conversion of H_2_O and CO_2 _into H_2_CO_3_, which then is ionized into H^+ ^and HCO_3_^- ^[21-23]. Mutations in CAII can also cause osteopetrosis due to non-functional osteoclasts [21]. The HCO_3_^- ^ions are exchanged for Cl^- ^through an anion exchanger, AE2, located in the basolateral membrane, leading to continued availability of Cl^- ^for acidification of the resorption lacuna [24-26].

Osteoclasts produce proteases, of which cysteine proteinase cathepsin K has proven to be the most important [27-30], aiding the degradation of the organic bone matrix. Osteoclasts secrete cathepsin K into the resorption lacunae, resulting in type I collagen matrix cleavage [27-30]. Cathepsin K gives rise to specific degradation products like C-terminal cross-linking telopeptide of type I collagen (CTX-I), which can be used for measurements of bone resorption [31]. Lack of cathepsin K results in pycnodysostosis, a disease characterized by high bone mass and skeletal deformities [27-30]. Matrix metalloproteinases (MMPs) have also been shown to participate in bone resorption [32-34], during which, MMP activity is known to give rise to a specific degradation fragment, C-terminal telopeptide of type I collagen (ICTP) [31]. The roles of MMPs appear, however, to be dependent on the type of bone the osteoclasts are to resorb, as MMP activity have been shown to have higher importance for resorption of flat bones compared to long bones [35-38]. Further complicating the understanding of the precise action and roles of these proteases is the fact that under some circumstances the activity of MMPs is masked by the activity of cathepsin K [33].

In this study we investigated the function of human osteoclasts in response to inhibited degradation of either the organic or inorganic bone matrix. For inhibition of acidification two independent V-ATPase inhibitors, bafilomycin A1 [39] and diphyllin [40], were used. A third acidification blocker used was ethoxyzolamide, inhibiting CAII [41]. For inhibition of organic resorption the broad-spectrum cysteine proteinase inhibitor, E64 [42], and the broad-spectrum MMP inhibitor, GM6001 [33], were used. Finally, ibandronate, a nitrogen-containing bisphosphonate [43], was tested to evaluate the effect of general osteoclast inhibition. Significant differences in the resorption profile of osteoclasts were seen in response to application of the different types of inhibitors.

## Methods

### Compounds

Bafilomycin was purchased from Tocris, diphyllin was purchased from Bioduro, E64 was purchased from Calbiochem, GM6001 was purchased from AM Scientific, and ibandronate and ethoxyzolamide were purchased from Sigma-Aldrich. All the remaining chemicals were from Sigma-Aldrich and the culture media were from Invitrogen, unless otherwise specified.

### Isolation of CD14+ human monocytes and osteoclast differentiation

CD14+ human monocytes were isolated as previously described [44]. Briefly, the monocytes were separated from human peripheral blood by centrifugation on a Ficoll-Paque gradient (Amersham Pharmacia), and magnetically sorted using a CD14+ magnetic bead isolation kit (Dynabeads M-450, Dynal Biotech). The cells were seeded in 75 cm^2 ^flasks and cultured in minimum essential medium alpha (αMEM) containing 10% fetal bovine serum, 100 units/ml penicillin, 100 μg/ml streptomycin, 25 ng/ml of macrophage colony stimulating factor (M-CSF) (R&D Systems) and 25 ng/ml receptor activator of NFκB ligand (RANKL) (R&D Systems) for 10-12 days.

### Osteoclast culture

When multinucleated (>3 nuclei), actin-ring positive osteoclasts were dominant in the differentiated osteoclast cultures (10-12 days after seeding CD14+ isolated monocytes), the mature osteoclasts were lifted for experiments as previously described by Sorensen et al. [44]. The mature osteoclasts were seeded on cortical bovine bone slices (50.000 cells/bone slice), and cultured in the presence or absence of inhibitors.

Bafilomycin was tested in the concentration range [0-30 nM], diphyllin in the concentration range [0-100 nM], ethoxyzolamide in the concentration range [0-100 μM], E64 in the concentration range [0-5 μM], GM6001 in the concentration range [0-100 μM], the combination treatment of E64 and GM6001 was tested in the concentration ranges [0-(5 μM E64) + (100 μM GM6001)] and ibandronate was tested in the concentration range [0-100 μM]. The choice of specific concentration ranges for the individual inhibitors was based on previous studies [6,33,36,40,44-46].

Medium was exchanged at day 2 after seeding. Supernatants from day 5 were collected, and the various resorption markers were measured in these.

All experiments with each of the individual inhibitors were conducted three times or more. The presented data are from one representative experiment for each inhibitor. Number of replicates in each experiment equals five.

For further investigations of supernatants and resorbed bone slices, the specific concentrations tested for the individual inhibitors were chosen based on maximum inhibition of bone resorption, without significant decrease in cell viability.

### Cortical bovine bone slices

Bone slices were cut from bone sticks (Nordic Bioscience A/S), which originated from femoral cortical bone from cows. The sticks were cut into slices with a thickness of 0.2 mm. The bone slices, which fitted into 96-wells, had a diameter of approximately 6 mm.

### Measurements of resorption

Organic bone resorption was measured by the release of both CTX-I and ICTP during culture.

CTX-I concentrations in culture supernatants were determined using CrossLaps for Culture (IDS Nordic) according to the manufacturer's instructions.

Concentrations of ICTP were measured using the ICTP EIA kit (Orion Diagnostica Denmark A/S) according to the manufacturer's instructions. For background measurements, medium from bone slices without cells was utilized. To estimate low ICTP concentrations, an alternative standard curve to the suggested was generated, using linear regression for the lowest concentrations in the standard row.

The concentration of total calcium, as an indicator of inorganic bone resorption, was measured in culture supernatants utilizing an ADVIA 1800 machine (Siemens). The principle of this measurement is a colorimetric assay in which the compound Arsenazo III forms a purple complex with calcium under acidic conditions. For background measurements, medium from bone slices without cells was utilized.

### TRACP activity

Tartrate resistant acid phosphatase (TRACP) activity was used as an indirect measure of osteoclast number [47,48]. TRACP activity was measured as previously described [6]. Briefly, samples were incubated with TRACP reaction buffer containing *p*-nitrophenyl phosphate and sodium tartrate [6], for 1 hour at 37°C in the dark. Reaction was stopped with the addition of 0.3 M NaOH. Absorbance was measured in an ELISA reader at 405 nm with 650 nm as the reference.

### Pit staining and counting

Bone resorption was evaluated by examinations of the resorbed bone area. Mature osteoclasts were cultured for five days on bone slices after which the adherent cells were removed using a cotton swab. The bone slices were washed in distilled water, and pits were stained with Mayer's hematoxylin (Bie & Berntsen A/S), followed by washing in water and removal of excess dye with a cotton swab. The resorbed area was measured using CAST-Grid (Olympus). The results were shown as resorbed area relative to the total area of bone. Pictures of the stained resorption pits were taken with an Olympus DP71 camera (x10 magnification).

### Cell viability

To assess cell viability, Alamar blue measurements were performed according to the manufacturer's protocol (Trek Diagnostics Systems). Briefly, Alamar blue was diluted 1:10 in the cell culture medium, and the color change was monitored carefully. When a switch from blue to purple was observed, the color changes were measured using a plate reader (excitation wavelength 540 nm, emission 590 nm). Alamar blue-containing medium added to wells without cells was used as background.

### Gelatinase zymography

MMP activity in culture supernatants was tested by gelatinase zymography as previously described [49]. Briefly, samples were run on a SDS-PAGE with a gelatin gel. The gel was subsequently incubated for 2 days in a 1% Triton X-100 buffer to wash out the SDS and to restore enzyme activity. The gel was stained with coomassie blue followed by de-staining and pictures were taken. MMP-9 was used as a positive control.

Three individual gels were run and average MMP-9 band intensities were analyzed using ImageJ.

### Calculation of IC50 values

The IC50 values for individual inhibitors were calculated using Prism 3.0. For these calculations the vehicle was set to 100% and all other data were represented as % of vehicle plotted against log[Treatment]. IC50 values were calculated using a nonlinear regression curve fit.

### Statistics

Statistical analyses were performed using one-way analysis of variance (ANOVA) followed by Dunnett's multiple comparison tests to adjust for multiple statistical testing against the control group. Statistical significance is indicated by the number of asterisks, p < 0.05 = *, p < 0.01 = ** and p < 0.001***. Error bars on all graphs indicate standard error of mean (SEM). Relative intensities of bands in gelatinase zymography gels are given ± standard deviation (SD).

## Results

### Inhibition of acidification results in equivalent inhibition of calcium release and collagen degradation

**Figure 1:**
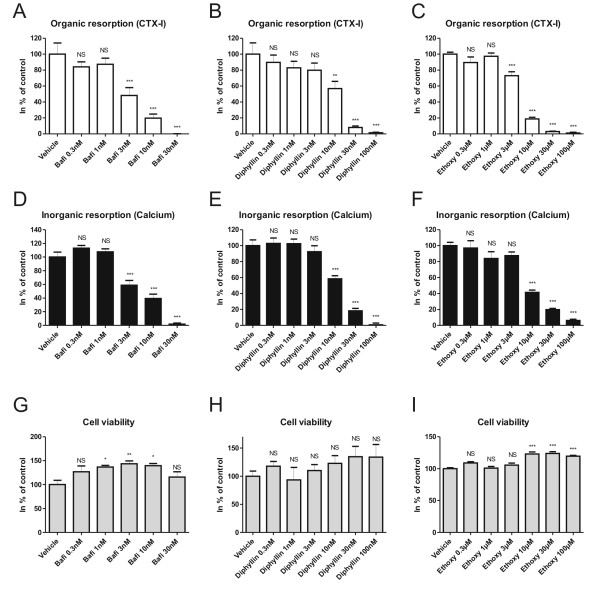
**The effect of inhibiting the acidification processes during osteoclastic resorption**. Mature human osteoclasts were seeded on bone slices and cultured for 5 days in the presence or absence of bafilomycin A1 (Bafi), diphyllin or ethoxyzolamide (Ethoxy). A-C) CTX-I release measured in culture supernatants. D-F) Calcium release measured in culture supernatants. G-I) Cell viability measured by Alamar Blue. All results are plotted as % of control. Statistical tests were performed by ANOVA with Dunnet's multiple comparison test against the vehicle group.

All three acidification inhibitors decreased the ability of mature osteoclasts to resorb both inorganic and organic bone matrix. As seen in Figure 1, bafilomycin A1, diphyllin, and ethoxyzolamide all inhibited bone resorption completely at the highest doses tested (bafilomycin 30 nM, diphyllin 100 nM and ethoxyzolamide 100 μM). The IC50 values for inhibition of organic and inorganic resorption for each of the three inhibitors are shown in Table 1. From these results it is apparent that all three acidification blockers inhibited the organic and inorganic bone matrix degradation equally well (Table 1 and Figure 1A-F). Cell viability varied only slightly, which was unrelated to the effects on bone resorption (Figure 1G-I). Apart from a decrease at the highest dose of diphyllin (100 nM) and bafilomycin (30 nM), no other reduction in TRACP activity was observed, indicating that mature osteoclasts were present (data not shown).

**Table 1:** IC50 values for each acidification blocker on inhibition of organic and inorganic resorption

Treatment	Organic resorption (CTX-I)	Inorganic resorption (Calcium)
**Bafilomycin**	3.1 nM	5.7 nM

**Diphyllin**	9.1 nM	12.4 nM

**Ethoxyzolamide**	4.9 μM	8.8 μM

**Figure 2:**
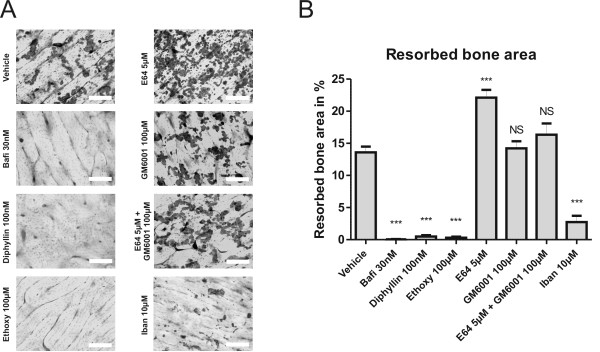
**Evaluations of resorbed bone area by different types of osteoclast inhibitors**. Mature human osteoclasts were seeded on bone slices and cultured for 5 days in the presence or absence of the indicated inhibitors. A) Pits were stained and pictures of the indicated conditions were taken. B) The stained pits were counted using CAST grid. Percent resorbed bone area was calculated and plotted. Statistical tests were performed by ANOVA with Dunnet's multiple comparison test against the vehicle group. Scale bars = 200 μm.

As seen in Figure 2A and 2B, all the acidification blockers at the highest concentrations tested reduced the resorbed bone area dramatically, as expected from the reduced organic and inorganic bone resorption seen in Figure 1 and previous studies [23,40,41,50].

### Inhibition of cathepsin K potently reduces organic and modestly decreases inorganic resorption but increases resorbed bone area

**Figure 3:**
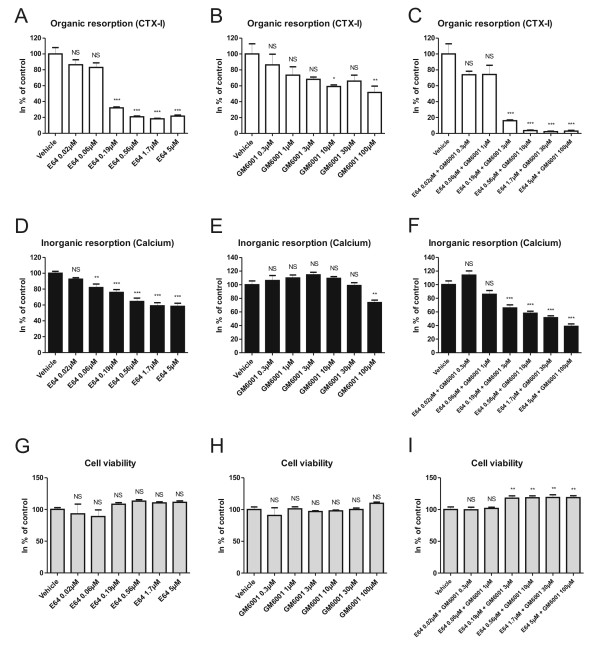
**The effect of inhibiting proteolysis during osteoclastic resorption**. Mature human osteoclasts were seeded on bone slices and cultured for 5 days in the presence or absence of E64, GM6001 or a combination of E64 and GM6001. A-C) CTX-I release measured in culture supernatants. D-F) Calcium release measured in culture supernatants. G-I) Cell viability measured by Alamar Blue. All results are plotted as % of control. Statistical tests were performed by ANOVA with Dunnet's multiple comparison test against the vehicle group.

E64, which inhibits cathepsin K, potently reduced organic resorption by approximately 80% at concentrations of 0.56-5 μM (Figure 3A). In contrast, inorganic resorption was reduced by approximately 40% in the same concentration range (Figure 3D). Cell viability measurements showed that the reduction in organic resorption was not due to toxicity (Figure 3G), and measurements of TRACP activity showed no decrease in the number of mature osteoclasts (data not shown). As seen in Figure 2A and 2B, the resorbed bone surface for cells treated with E64 at a concentration of 5 μM was significantly higher than for vehicle-treated cells.

### MMP-mediated bone degradation is a minor but normal part of bone resorption

Use of GM6001 to inhibit MMP-mediated resorption produced a modest reduction in bone resorption of both organic and inorganic matrix, but only at the highest concentration of the inhibitor (100 μM) (Figure 3B and 3E). This effect was not caused by decreased cell viability or decreased number of osteoclasts as measured by TRACP activity (Figure 3H, and data not shown). When both cathepsin K and MMP-mediated bone resorption was inhibited, CTX-I release was completely abrogated at concentrations: E64 [0.56 μM] + GM6001 [10 μM] and above, indicating that both cathepsin K and MMP activities are important for bone resorption, with cathepsin K playing the major role (Figure 3A-C). Measurements of cell viability showed that decreased cell viability was not the cause of decreased resorption (Figure 3I), but a slight decrease in TRACP activity was seen after combination treatment with the highest doses of E64 [5 μM] and GM6001 [100 μM] (data not shown). A more pronounced reduction in inorganic resorption was seen with simultaneous treatment with E64 and GM6001 than after either treatment alone (Figure 3D-F). This reduction in inorganic resorption, however, did not match the reduction in organic resorption, with the IC50 value for organic resorption being 20 times higher than for inorganic resorption. Treatment with GM6001 at 100 μM did not change the extent of resorbed bone area compared with the area associated with vehicle-treated cells (Figure 2A and 2B), which is in alignment with previously published data [13]. In addition, following co-treatment of cells with E64 [5 μM] and GM6001 [100 μM], the percentage of resorbed bone area was not significantly different from that seen with vehicle-treated cells (Figure 2B).

### MMPs appear to compensate for the absence of cathepsin K

**Figure 4:**
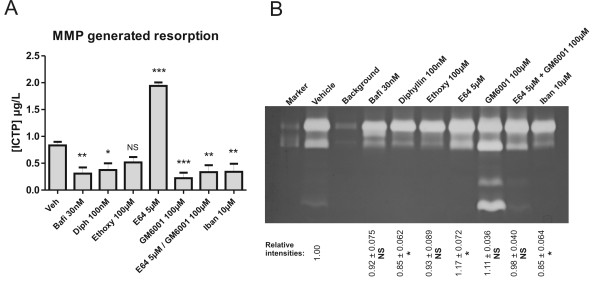
**Evaluation of MMP generated resorption and MMP secretion**. Mature human osteoclasts were seeded on bone slices and cultured for 5 days in the presence or absence of the indicated inhibitors. A) ICTP levels were measured in culture supernatants. B) Gelatinase zymografi of pooled culture supernatants. MMP-9 is used as marker. A representative gel is shown. Based on analysis of three individual gels in ImageJ, relative band intensities, reflecting MMP-9 activity, were calculated. Relative intensities are given ± SD.

In the vehicle-treated condition a minor release of ICTP fragments was observed (Figure 4A), indicating that a low level of MMP-mediated resorption took place during normal resorption. All the tested inhibitors, except E64, had either no effect or inhibited the release of ICTP, with GM6001 showing the most potent reduction (Figure 4A). In contrast, inhibition with E64 strongly increased the ICTP release (Figure 4A). Gelatinase zymography of culture supernatants of all tested inhibitors was performed to evaluate MMP activity. As seen in figure 4B, MMP-9 activity was significantly increased with E64 treatment. With all other treatments, MMP-9 activity was either unaltered or significantly lowered (diphyllin and ibandronate) in comparison with vehicle (Figure 4B).

During treatment with GM6001, the osteoclasts responded by secreting more types of MMPs (Figure 4B). However, this was not followed by an increase in ICTP release (Figure 4A).

### Ibandronate potently inhibits both organic and inorganic resorption by reducing the number of mature osteoclasts

**Figure 5:**
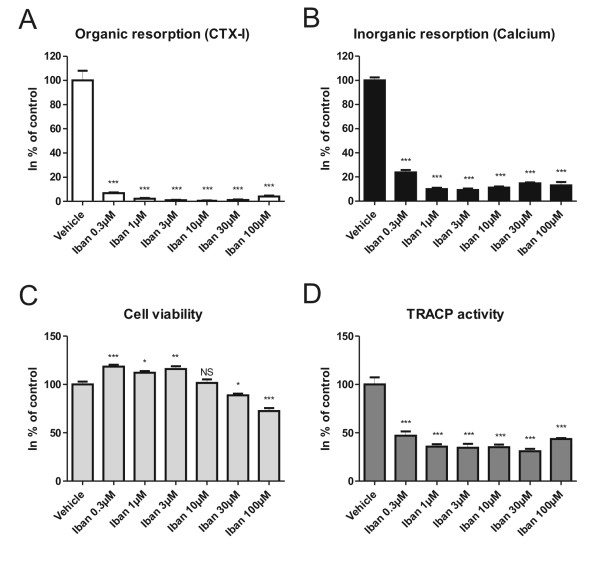
**The effects of ibandronate on osteoclasts and resorption**. Mature human osteoclasts were seeded on bone slices and cultured for 5 days in the presence or absence of ibandronate (Iban). A) CTX-I release measured in culture supernatants. B) Calcium release measured in culture supernatants. C) Cell viability measured by Alamar Blue. D) TRACP activity measured in culture supernatants. All results are plotted as % of control. Statistical tests were performed by ANOVA with Dunnet's multiple comparison test against the vehicle group.

As seen in figure 5A and 5B, the bisphosphonate, ibandronate, strongly inhibited both organic and inorganic bone resorption at all concentrations tested [0.3-100 μM]. This correlated with the strong reduction in the percentage of resorbed bone area (Figure 2A and 2B). Cultured cells showed decreased viability when treated with ibandronate at concentrations of 30 μM and above, but at concentrations from 0.3-10 μM cell viability was unaffected or even slightly increased (Figure 5C). TRACP activity was, however, strongly decreased at all concentrations tested, indicating a strong reduction in the number of mature osteoclasts.

## Discussion

We investigated the differences and similarities between inhibition of acidification and proteolysis as well as general osteoclast inhibition, with respect to the resorptive phenotype of the osteoclasts. We, for the first time, present data suggesting that different classes of bone resorption inhibitors may produce different phenotypes of human osteoclasts. We speculate that these divergent phenotypes in some aspects may reflect osteoclast quality, and may have secondary effects on bone quality and bone formation through the coupling process. In the present study, human osteoclasts derived from CD14+ monocytes were utilized, although it must be noted that osteoclasts from other sources, for example bone marrow, might have given different resorption profiles [38], which could be of interest to study in the future.

**Figure 6:**
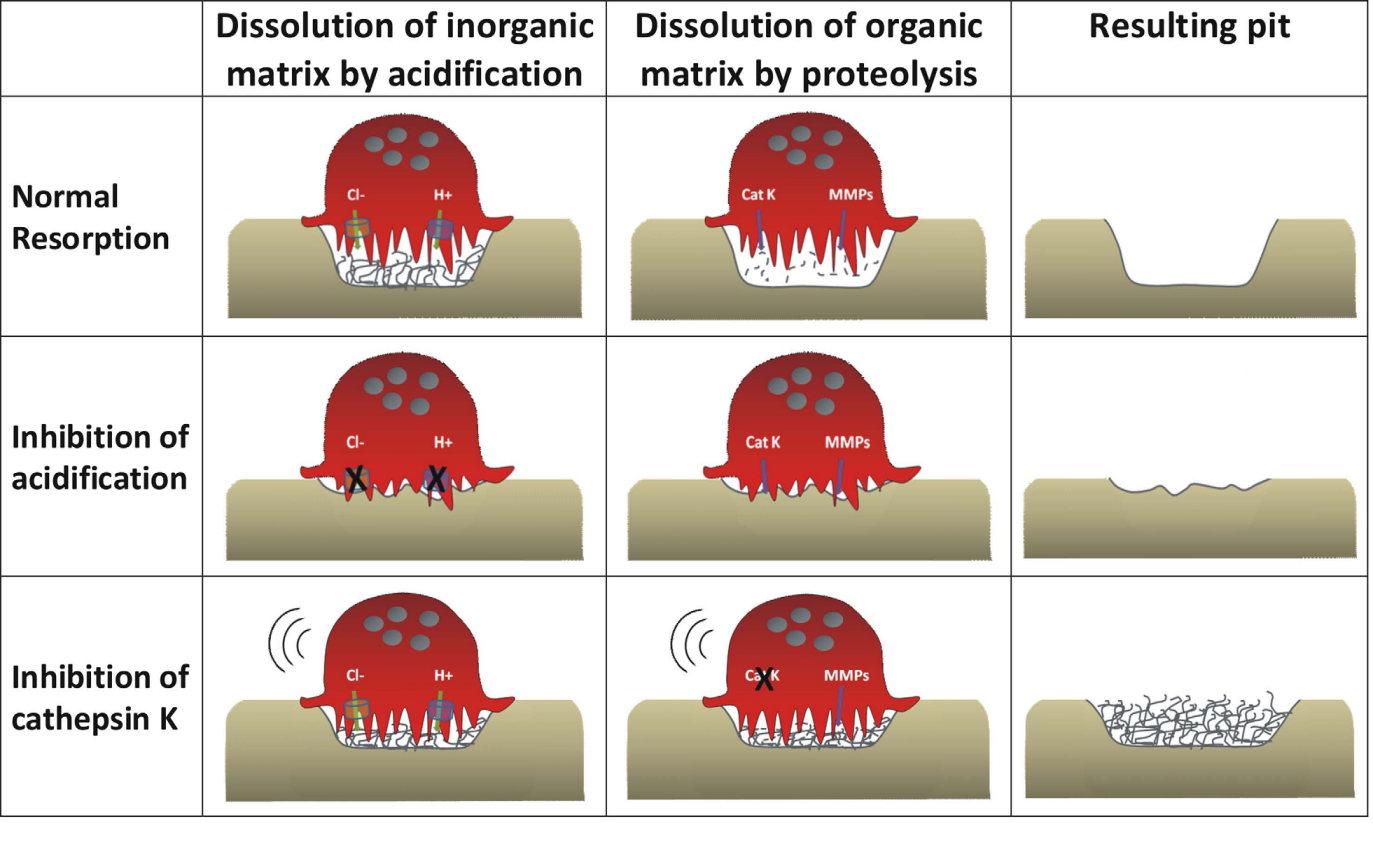
**Schematic illustration of normal or inhibited osteoclastic resorption**. Upper panel: Normal bone resorption with acidification of the resorption lacuna followed by proteolysis for removal of the organic matrix. The resulting pit appearance is shown in the column to the far right. Middle panel: Inhibition of acidification leads to disruption of both organic and inorganic resorption. Only residual pits are formed. Lower panel: Inhibition of cathepsin K will not prevent inorganic resorption, whereas organic resorption is potently inhibited. The resulting pits will be packed with non-dissolved organic matrix, and the pits will have a lower depth than normal pits.

Bafilomycin A1 and diphyllin, both inhibitors of the V-ATPase, and ethoxyzolamide, a CAII inhibitor, were all used as inhibitors of acidification within the resorption lacuna. All three compounds completely abrogated bone resorption measured by all parameters (CTX-I, calcium, ICTP and resorbed bone area). In addition, IC50 values for inhibition of organic and inorganic bone resorption were found to be equal for the individual inhibitors of acidification (Table 1). Thus, inhibiting the acidification processes attenuates bone resorption, without the possibility of potential compensatory mechanisms *in vitro *(Figure 6, middle panel). This is in alignment with previous publications [6,40,51].

IC50 values were not calculated for the proteolysis blockers as these inhibitors individually did not inhibit bone resorption completely. Instead, the maximum level of inhibition of resorption was investigated. For treatment with E64 inhibiting cathepsin K, organic resorption was reduced to a level of 20% in comparison to vehicle-treated osteoclasts, whereas inorganic resorption was reduced to a level of 60%. This is in line with the role of cathepsin K in degradation of the organic bone matrix [29] (Figure 6, lower panel). A similar phenomenon was observed after inhibition of MMP activity with GM6001, although the overall level of inhibition was markedly lower than for E64 treatment. As both cathepsin K and MMPs are important for degradation of the organic matrix, it can be speculated why inhibitors of these enzymes also affect resorption of the inorganic matrix. In the process of bone resorption, acidification and proteolysis cannot be considered as two separate processes. Under normal circumstances the two processes will occur simultaneously, and if organic resorption is hindered, the osteoclasts will not be able to dig as deep into the bone as under normal bone resorption (Figure 6, lower panel) [42,52], which will lead to the observed decrease in inorganic resorption. In line with this, studies of bone organ cultures showed that E64 reduced Ca^2+ ^release as well as the expected reduction in organic resorption [31,53].

Interestingly, we observed that the levels of MMP-generated type I collagen fragment, ICTP, were elevated when cathepsin K activity was reduced, whereas the other inhibitors either left the ICTP level unchanged in comparison to vehicle, or lowered the level. These data are in line with *in vivo *findings in pycnodysostotic patients, who have abnormally high levels of ICTP, and with previous *in vitro *studies in which E64 was utilized [28,33]. It should, however, be noted that the basal level of ICTP release seen in the vehicle condition is in contrast to previously obtained data [33]. The reason for this discrepancy could be the bone source, as bone of various origin are resorbed differently [32,36,37]. However, a more likely explanation is the fact that the levels of ICTP, except for E64-treated cells, were close to the detection limit and thus hard to quantify. These data indicate that in the absence of cathepsin K activity, MMPs may perform compensatory collagen cleavage, which has previously been suggested [28,33]. However, as ICTP fragments are destroyed by cathepsin K [31,54], the MMP turnover profile of type I collagen may be masked under normal physiological bone turnover. However, increased MMP activity for the E64-treated condition was observed, which indicates that a compensatory MMP-generated bone resorption did take place. Thus, it appears that the increase in ICTP levels is a combination of both an unmasking and a compensatory effect. These data also indicate that the effect of cathepsin K inhibitors may be overestimated when using CTX-I or other cathepsin K-generated collagen fragments, such as NTX. These collagen fragments specifically monitor the enzymatic activity of cathepsin K [55] and do not reveal other compensatory organic resorption processes or the continued resorption of the inorganic matrix.

The increased area of resorbed bone observed for osteoclasts treated with E64 is rather surprising. Measurements of cell viability and TRACP activity, did not suggest that an increased number of osteoclasts was responsible for the increased resorbed bone area. Previous studies with E64 did not show an increase in osteoclast number either [42]. It is therefore speculated that the E64-treated osteoclasts are more motile, which could explain the increased resorbed area. An increased motility of E64-treated osteoclasts could potentially be explained by the increased MMP activity in these cells, as MMPs are known to be associated with cell motility [56]. Supporting this is the observation that GM6001 blunted the effect of E64 with respect to increased resorbed bone area, thereby suggesting that MMP activity indeed was the cause of the elevated resorbed bone surface observed for E64-treated osteoclasts.

It must, however, be clarified that bone resorption should be considered to be volumetric rather than areal, which signifies that an increased resorbed bone surface does not correlate with increased bone resorption, as this parameter is clearly lowered for the E64-treated osteoclast. As both organic and inorganic resorption was shown to be lowered by 5 μM E64, despite an increased resorbed bone area at the same concentration, the resorption pits are likely more shallow than normal resorption pits, which is in line with previous studies [42,52].

The present findings of an increased resorbed bone area in the presence of E64 correlate well with *in vivo *findings in mice, as well as in patients with pycnodysostosis, arising from cathepsin K deficiency, where demineralized areas are observed below the osteoclasts [28,29]. This could also explain why a marked decrease in the bone resorption markers CTX-I and NTX, by 55-70%, was seen in cathepsin K inhibitor trials, whereas the effect on BMD often was less marked (2.5-5.7%) [57,58]. The finding of increased resorbed bone area with E64 treatment is, however, in contrast to a recent study by Ainola et al. [59]. This might be due to changes in cellular behavior dependent on the culture substrate, as our study was performed with cortical bone slices whereas the other study was conducted with dentine [59].

In our study, ibandronate was found to be a potent inhibitor of both organic and inorganic bone resorption. Ibandronate is a nitrogen-containing bisphosphonate which, like alendronate and risedronate, will bind strongly to the bone matrix [60-62]. Under acidic conditions, induced by the osteoclasts in the resorption lacuna, the bound bisphosphonate will be released from the matrix and in this way target the osteoclasts specifically [60-62], inducing reduced resorptive activity and apoptosis [63,64]. On the other hand, non-resorbing cells, like macrophages, have been shown not to be greatly affected by bisphosphonate treatment on dentine, as the cells are not able to release and take up substantial amounts of the drug [62]. Ibandronate treatment in our study was shown to reduce TRACP activity dramatically for all concentrations tested [0.3-100 μM]. This strongly indicates that the number of mature osteoclasts is reduced dramatically in these cultures, as strong correlations between TRACP activity and osteoclast numbers have been reported [47,48]. However, the overall cell viability of the cultures was not decreased for most of the tested concentrations. The discrepancy between low TRACP activity and normal cell viability can be explained by the presence of macrophages in the culture. When mature osteoclasts are seeded on bone slices, a portion of these cells will be macrophages [65], which will either proliferate or differentiate into mature osteoclasts. As the macrophages are not greatly affected by bisphosphonate treatments when cultured on bone [62], this could explain the sustained cell viability despite a decrease in the number of osteoclasts. Risedronate, another nitrogen-containing bisphosphonate, has been shown to strongly inhibit osteoclastogenesis *in vitro *[66], which could also be the case for ibandronate, partly explaining the low TRACP levels. If osteoclast differentiation is inhibited, an increased number of macrophages would be expected, which could uphold the cell viability measure. In addition, nitrogen-containing bisphosphonates are known to induce osteoclast apoptosis [63,64], thereby reducing the osteoclast number. However, the anti-resorptive effect of nitrogen-containing bisphosphonates has been shown to take place at concentrations that are lower than for osteoclast apoptosis [64]. This could indicate that at lower concentrations, osteoclast apoptosis is not induced, despite attenuation of the resorptive activity. Nitrogen-containing bisphosphonates inhibit farnesyl diphosphate (FPP) synthase, which attenuates osteoclast function, in part by disrupting vesicular transport [63,67]. As release of TRACP from mature osteoclasts is dependent on vesicular trafficking [68], the reduced TRACP activity might also reflect this issue at concentrations where osteoclast-specific apoptosis is not induced. The three scenarios: decreased osteoclastogenesis, osteoclast apoptosis and obstruction of TRACP release, could therefore all potentially be involved in the reduction of TRACP activity, and it would be of interest to examine these aspects further. However, regardless of the mechanism for lowering the osteoclast number or attenuation of resorptive activity, the resorptive profile is clear: both organic and inorganic bone resorption are abrogated at ibandronate concentrations of 1 μM and higher (Figure 5).

Recent studies have indicated that the osteoclast phenotype is important for the control of bone remodeling [4,7]. Osteoclasts likely control bone formation both directly by secretion of molecules with anabolic effects on osteoblasts, but also through the modulation of the surface of the resorption pits, which is dramatically altered when cathepsin K activity is absent [4,7,8]. The different mode of actions of the utilized osteoclast inhibitors can therefore be speculated to have different treatment profiles when used as, for example, treatments of osteoporosis. All anti-resorptive drugs currently used in clinical settings display reduced fracture risks due to abrogation of osteoclast functionality, which leads to lower activation frequency of new remodeling sites. However, differences between treatments exist. Bisphosphonates are associated with a big drop in osteoclast number, strongly reduced bone resorption and secondarily a strong reduction in bone formation [69]. A recent study with odanacatib, a specific cathepsin K inhibitor, has demonstrated a strong decrease in bone resorption, an initial drop in bone formation, which subsequently increased towards the baseline level [58]. This indicates that in this type of treatment, a strong coupling between resorption and formation is not seen as in the case of bisphosphonate treatments [58]. This could potentially be due to maintenance of osteoclasts within the bones when cathepsin K is targeted, in contrast to treatments with bisphosphonates. However, the clinical study with odanacatib demonstrated that the resorption surface was unaltered in the treatment groups in comparison to the placebo group [58]. This finding, that initiation of bone resorption is continued despite inhibition of cathepsin K, was confirmed in the present study. No clinical trials have so far been conducted with acidification inhibitors, however, animal studies demonstrate that bone resorption can be lowered with these agents, without a subsequent lowering of bone formation [51]. Furthermore, it can be speculated that treatment with acidification blockers could lead to a higher increase in BMD, in comparison to treatments with cathepsin K inhibitors, as resorption of the inorganic matrix is prohibited.

## Conclusions

In conclusion, we examined the osteoclast phenotype in response to inhibition of acidification, proteolysis or general osteoclast inhibition. We found that inhibition of acidification, leading to inhibited dissolution of the inorganic bone matrix, completely shut down the ability of osteoclasts to resorb bone as a whole. In contrast, inhibition of the cysteine proteinases strongly reduced release of CTX-I, but might allow compensatory bone resorption by MMPs, and inorganic resorption was only modestly reduced. An illustration of our proposed mechanism of osteoclast action is given in Figure 6, illustrating both inhibition of acidification and proteolysis. Ibandronate was shown to be a potent inhibitor of all aspects of bone resorption, by specifically reducing the number of mature osteoclasts. Taken together, bone resorption inhibitors may induce different osteoclast phenotypes that may be associated with different osteoclast qualities. As osteoclast number and quality are believed to be important for bone quality, investigation and understanding of these parameters for osteoporosis intervention therapies may provide additional information and understanding of the effect on skeletal health.

## Abbreviations

αMEM: Minimum essential medium alpha; ADOII: Autosomal dominant osteopetrosis type II; ANOVA: One-way analysis of variance; ARO: Autosomal recessive osteopetrosis; CAII: Carbonic anhydrase II; CTX-I: C-terminal cross-linking telopeptide of type I collagen; FPP: Farnesyl diphosphate; ICTP: C-terminal telopeptide of type I collagen; M-CSF: Macrophage colony stimulating factor; MMPs: Matrix metalloproteinases; RANKL: Receptor activator of NFκB ligand; SD: Standard deviation; SEM: Standard error of mean; TRACP: Tartrate resistant acid phosphatase; V-ATPase: Vacuolar ATPase

## Competing interests

Morten A. Karsdal is employed by and owns stocks in Nordic Bioscience.

The following persons are currently employed by Nordic Bioscience but own no stocks in the company: Anita V. Neutzsky-Wulff, Mette G. Sorensen, Diana J. Leeming and Kim Henriksen.

All other authors have no conflicts of interest.

## Authors' contributions

AVN designed the resorption experiments, measured resorption markers, performed gelatinase zymography and drafted the manuscript. DJL and DK conducted the pit staining and counting of pits. MHD was responsible for collection of the human blood samples. MGS, MAK and KH participated in the experiment design and have helped to draft the manuscript. All authors have contributed with inputs and have read and approved the final manuscript.

## Pre-publication history

The pre-publication history for this paper can be accessed here:

http://www.biomedcentral.com/1471-2474/11/109/prepub
